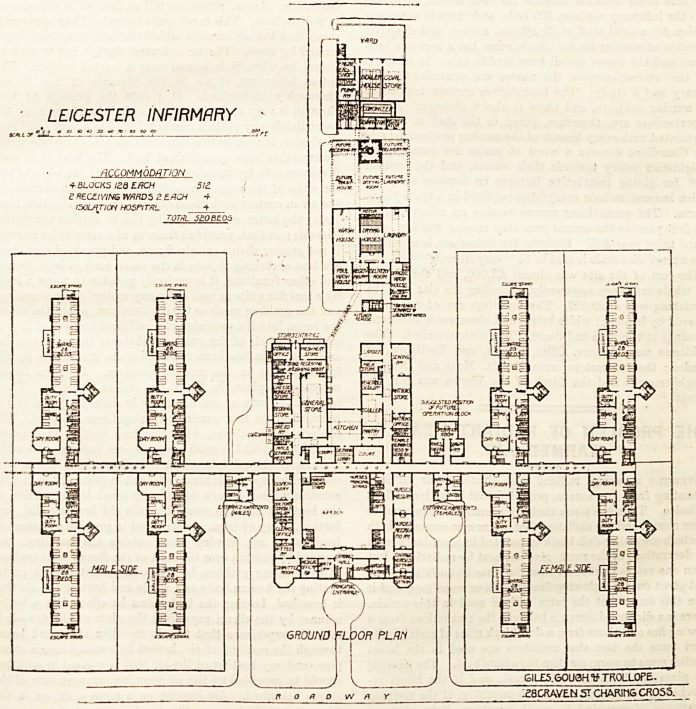# New Poor-Law Infirmary, Leicester

**Published:** 1906-04-21

**Authors:** 


### NEW POOR-LAW INFIRMARY, LEICESTER.

The site obtained by the Guardians for this hospital is on Crow Hill, about two miles from the centre of Leicester, and is sixty-two acres in extent. The foundation stone of the building was laid on April 2, 1903, by Mr. A. Kemp, who is, or was, chairman of the Board; and the hospital was opened by the same gentleman on September 28, 1905, in presence of the Mayor of Leicester, several members of Parliament, town councillors and Guardians. The site is one which commands fine views of the neighbouring scenery.

In general plan the hospital is very simple. The pavilions and accessory departments are arranged on both sides of a long corridor which runs north-east and south-west. This corridor, which is about eight feet wide, has a dado of white tiles about four feet six inches high, with a blue border. In the exact centre of the corridor is placed the administrative department. On the left on entering the hall is the porter's room. Passing through the hall and going along the quadrangle passage we come to the waiting-room, medical officer's room, committee-rooms, clerk's office, fire-proof room, doctor's consulting-room, and the dispensary. Entering the main corridor we find an escape staircase in the angle, and at the opposite angle is the nurses' principal staircase, then the mess-room pantry, and nurses' lavatory. We are now in the quadrangular passage again, and exactly opposite the staircase and other rooms just mentioned is the nurses' mess-room. This room is about forty feet long, and it looks as if the corresponding part of the quadrangular passage might be a little dark. Next the nurses' mess-room is the nurses' recreation-room, then the charge nurses' sitting-room, and the matron's sitting-room. From this point a staircase leads to the first floor, and next the staircase is the nurses' visitors' room, then the library, the office, and the entrance hall again.

At the other side of the main corridor and in the centre is the serving lobby, having on each side an open court, and behind it is the kitchen, with the pantry, scullery, vegetable-room, milk-store, and larder. Excepting the vegetable scullery these rooms are approached by a roof-lighted passage having on its other side the servants' mess-room, matron's store and serving-room. The passage is continuous with a covered way which opens into a well-arranged laundry, the machinery in which is driven by electric power. There is ample space behind the laundry for future extension, if that should at any time be desirable, and behind this vacant space is the power-house. Returning to the main corridor, we enter another roof-lighted passage similar to the one already named, and this has on one side the general stores and the meat store, and on the other side the steward's office, the staircase, sample room, cheese room, bedding store, bread room, and male servants' pantry and mess-room. The first floor of this department (on both sides of the main coridor) is given up to bedrooms and other accommodation for the officers, nurses, and domestic servants. The front part only of this block is carried up to a second floor, so as to increase the number of rooms for nurses. Altogether, there are rooms for forty-five nurses. Speaking generally it may be said that the administrative block is very well designed; and, with the possible exception named, there would seem to be plenty of light and air throughout it. The department bears a considerable resemblance to the administrative departments of several of our county asylums, in which institutions the needs of a large staff have been carefully worked out and the accommodation required as carefully evolved, so that they are safer models to copy than are general hospitals.

Passing along the main corridor for about twenty feet towards the female side we reach the entrance for female patients. This little block contains waiting-room, bath-room, closet, etc., and a two-bedded ward. On the opposite side of the corridor is, or will be, placed the operating theatre with its adjuncts. In another twenty feet or so we get to one of the pavilions. These pavilions are two stories high, and each floor is divided into two separate wards. These wards are practically alike, so that a descrip-

### LEICESTER INFIRMARY.

FIRST FLOOR PLAN.

tion of one will enable the reader to understand the planning of them all on both sides of the infirmary. Turning out of the main corridor to the left we have on the left-hand side the main staircase. Next this staircase is the day-room, the end of which is formed into a large bay window; then we have the three-bedded ward. This ward has four windows, so that each bed has a window on both sides, which is good; but the opposite wall has in it the fireplace and the door, hence the only cross-current which can be obtained in the three-bedded ward is through the open door, or through a fanlight, if there be one, and even then the ventilation is the end of the passage leading to the sanitary annexe. Still, it would seem certain that about forty-eight feet of this passage is not directly lighted, and, although we admit that an objection like this sometimes appears less marked in reality than it does on plan, we doubt whether the means of approach to a ward containing twenty-eight beds are the best obtainable. The sanitary block is conveniently placed and arranged, and it is properly cut off from the ward by a cross-ventilated passage. The block contains sinks and two closets, the latter being in proportion of one to sixteen patients.

into a corridor. This part of the plan is, therefore, faulty, and there is no possibility of cross-ventilation through the ward endways, because one end wall abuts on the day-room and the other on the nurses' duty-room. Returning to the entrance to the block we have, opposite the staircase, the lift, and, opposite the other rooms named there are a one-bedded ward, a store, a linen-room, a bath-room, and a passage which leads to the sanitary block. The one-bedded ward is subject to the same drawback as the three-bedded ward. Regarding the connecting passage it is lighted to some extent by the staircase windows and by the window at

The ward is about 90 feet long and about 26 feet wide, and would therefore allow nearly eighty superficial feet of floor space per bed, and the wall space per bed about six feet six inches. These measurements must be understood to be approximate as the plans published herewith are of small scale and have no dimensions marked on them. The ward contains twenty-eight beds, and there are eight windows on each side, the beds being arranged in pairs between the windows. The nearest point between any pavilion and any other part of the infirmary is about 50 feet, and the furthest point about 75 feet.

Each ward has two central stoves; but it is stated that the warming is really due to the radiators which are patented by the Atmospheric Steam Heating Company. We doubt the wisdom of this plan, and we hold that the wards of a hospital should have their chief and usual source of heat from open fireplaces, and that hot water or steam radiators should merely be "reserves" ready to be called on when the temperature falls greatly.

All the ventilators and all the taps are "keyed." Electric light is used throughout; but gas is also laid on in case of failure in the electric light supply.

There is an isolation hospital for four beds. Including this the infirmary contains 520 beds, and there is accommodation for a total staff of 70 officers, nurses, and domestic servants of various kinds. Each nurse has a separate bedroom, and the rooms are all furnished in oak. In addition to the recreation-room the nurses are provided with a library and a study. The bookshelves contain text-books on nursing subjects, and there is also a skeleton. Ample opportunities are, therefore, given to the staff to become acquainted with every branch of the nursing profession; and the Guardians deserve a word of praise for pursuing an enlightened policy towards their nurses, and the medical staff for giving instructive lectures on nursing subjects, which lectures include everything required in a three-years' course. The probationer nurses receive no salary during the first year, in the second year they receive £12 10s., and in the third year £15. Uniform for indoor use is supplied. The nurses' diet scale is said to be "very liberal."

The cost of the site was almost £7,000, and the cost of the whole infirmary apparently, including the site and the furnishing was £120,000. The buildings are of red brick relieved by bands of white brick, and there are white-stone dressings to windows and doors. The roofs are tiled. The architects were Messrs. Giles, Gough, and Trolloppe, of London; the contractors were Messrs. Moss and Son, of Loughborough; and the Clerk of the Works was Mr. H. George.

## Figures and Tables

**Figure f1:**
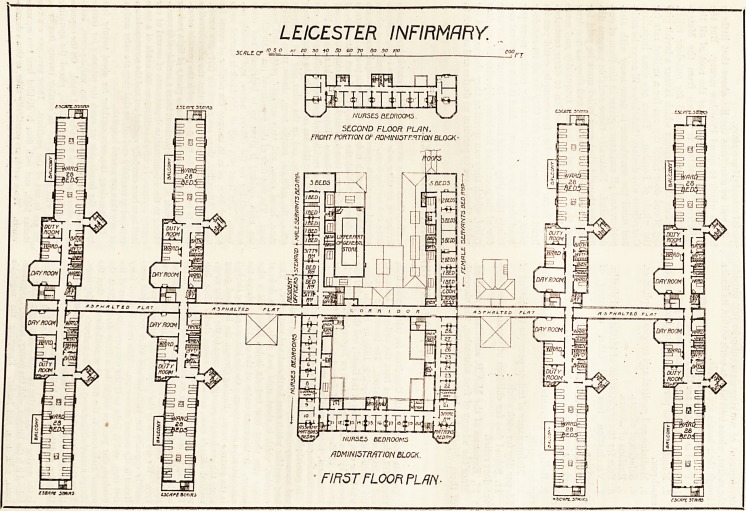


**Figure f2:**